# Two nights of recovery sleep restores hippocampal connectivity but not episodic memory after total sleep deprivation

**DOI:** 10.1038/s41598-020-65086-x

**Published:** 2020-05-29

**Authors:** Ya Chai, Zhuo Fang, Fan Nils Yang, Sihua Xu, Yao Deng, Andrew Raine, Jieqiong Wang, Meichen Yu, Mathias Basner, Namni Goel, Junghoon J. Kim, David A. Wolk, John A. Detre, David F. Dinges, Hengyi Rao

**Affiliations:** 1 Department of Psychology, Sun Yat-sen University, Guangzhou, China; 2 Laboratory of Applied Brain and Cognitive Sciences, School of Business and Management, Shanghai International Studies University, Shanghai, China; 3 Center for Functional Neuroimaging, Department of Neurology, University of Pennsylvania, Philadelphia, PA USA; 4 Division of Sleep and Chronobiology, Department of Psychiatry, University of Pennsylvania, Philadelphia, PA USA; 5 Center for Neuromodulation in Depression and Stress, Department of Psychiatry, University of Pennsylvania, Philadelphia, PA USA; 6 Department of Molecular, Cellular, and Biomedical Sciences, CUNY School of Medicine, The City College of New York, New York, NY USA; 7 University of Ottawa Brain and Mind Research Institute, Ottawa, Canada

**Keywords:** Cognitive neuroscience, Human behaviour

## Abstract

Sleep deprivation significantly impairs a range of cognitive and brain function, particularly episodic memory and the underlying hippocampal function. However, it remains controversial whether one or two nights of recovery sleep following sleep deprivation fully restores brain and cognitive function. In this study, we used functional magnetic resonance imaging (fMRI) and examined the effects of two consecutive nights (20-hour time-in-bed) of recovery sleep on resting-state hippocampal connectivity and episodic memory deficits following one night of total sleep deprivation (TSD) in 39 healthy adults in a controlled in-laboratory protocol. TSD significantly reduced memory performance in a scene recognition task, impaired hippocampal connectivity to multiple prefrontal and default mode network regions, and disrupted the relationships between memory performance and hippocampal connectivity. Following TSD, two nights of recovery sleep restored hippocampal connectivity to baseline levels, but did not fully restore memory performance nor its associations with hippocampal connectivity. These findings suggest that more than two nights of recovery sleep are needed to fully restore memory function and hippocampal-memory associations after one night of total sleep loss.

## Introduction

Insufficient sleep is a widespread problem in contemporary societies. Millions of people sleep less than 7 hours per night, which is the minimum sleep duration to prevent cumulative deterioration in neurobehavioral performance^1–5^. Sleep loss destabilizes the wake state, impairs cognition and behavior, increases the risk for multiple diseases, and incurs considerable social, financial, and health-related costs^6–9^.

Although the exact function of sleep remains to be elucidated, it is well known that sleep facilitates memory retention^10–13^, whereas sleep deprivation degrades memory performance and brain function^14–23^. Animal studies have consistently demonstrated the impairing effects of sleep loss on several neural circuits involved in learning and memory, in particular the hippocampal complex^16–18^. Accumulating evidence also suggests that newly encoded information temporarily stored in the hippocampus is reactivated during sleep and integrated with existing long-term memories stored in the neocortex^24–26^. In humans, sleep deprivation attenuates hippocampal function at rest as well as during memory encoding, consolidation, and recognition tasks^14,15,22,23,27^. A growing body of research suggests that functional connectivity between the hippocampus, prefrontal cortex, and default mode network (DMN) regions may play critical roles in memory function^28–32^, which are disrupted by sleep deprivation^15,20,22,23^.

Although the detrimental effects of sleep loss on memory and brain function are well established, whether and how recovery sleep restores memory and brain function after sleep loss are not well understood. To date, only a limited number of studies have examined the restoring effects of recovery sleep on cognitive and brain function. For example, an animal study suggested that several hours of recovery sleep restored hippocampal synaptic plasticity and connectivity in mice after brief sleep deprivation^33^. In humans, some studies have indicated that one or two nights of recovery sleep might be able to restore cognitive performance and brain adenosinergic system to baseline levels^34–40^, while others have suggested that neurobehavioral deficits, self-monitoring abilities, and brain metabolic decreases after total or chronic sleep loss might not be fully recovered by one or two nights of recovery sleep^1,3,41–46^. A recent study found that a 90-min recovery nap restored hippocampus-dependent learning during the day of sleep deprivation, and the structural morphology of hippocampal subfields predicted the success of learning restoration^47^. However, it remains unclear about how much amount of recovery sleep people would need to fully restore both cognitive and brain function following sleep deprivation.

**Figure 1:**
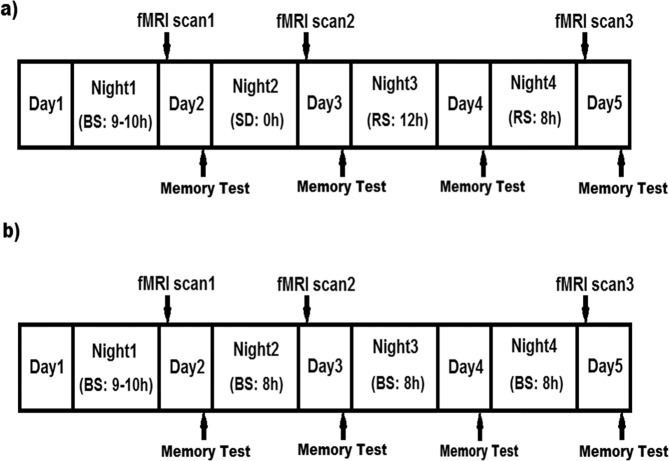
The 5-day and 4-night experimental protocols for (**a**) the total sleep deprivation (TSD) group, and (**b**) the non-sleep-deprived control group. Participants arrived at the laboratory in the afternoon of Day 1 and were provided 9–10 h time-in-bed (TIB) for baseline sleep (BS) on Night 1. After the first scanning session (fMRI scan 1) in the morning of Day 2, participants were randomized to either a TSD or a control condition. On Night 2, the TSD group was kept awake while the control group was allowed 8 h TIB control sleep. After the second scanning session (fMRI scan 2) in the morning of Day 3, the TSD group was then allowed 12 h TIB and 8 h TIB recovery sleep (RS) on Night 3 and Night 4 respectively, while the control group was allowed 8 h TIB control sleep on both nights. The third scanning session (fMRI scan 3) took place in the morning of Day 5. The scanning time for the three scanning sessions was kept constant from 0700 h to 1000 h, and each participant was scanned at the same time across the three scanning sessions to avoid the potential time-of-day differences (scan duration for each participant was about an hour). Participants completed the Memory Test in each afternoon during the protocol.

The present study used resting-state functional magnetic resonance imaging (fMRI) and aimed to examine both the impairing effects of one night of total sleep deprivation (TSD) and the restorative effects of two consecutive nights (12-hours and 8-hours time-in-bed) of recovery sleep following TSD on episodic memory and hippocampal functional connectivity in a well-controlled 5-day and 4-night in-laboratory study (see Fig. 1). We also studied an additional cohort of control participants using the same protocol but without sleep loss to exclude the potential confounding effects of in-laboratory experimental procedures on brain connectivity and memory performance. While a number of literature has demonstrated the alteration of brain connectivity in different resting-state networks after sleep deprivation^20–23,93^, we focused on the effects of recovery sleep following sleep deprivation on hippocampal functional connectivity and episodic memory performance in this study. Based on previous literature, we hypothesized that one night of acute TSD would significantly disrupt episodic memory performance (H1a) and the underlying hippocampal connectivity (H1b) as compared to baseline levels, whereas two nights of recovery sleep after one night of acute TSD would restore both episodic memory performance (H2a) and hippocampal connectivity (H2b) to baseline levels.

## Results

**Figure 2:**
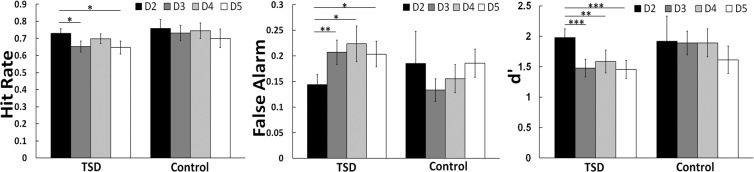
Averaged episodic memory performance (hit rate, false alarm, and discrimination scores (d’)) (±SEM) across the four consecutive days for the total sleep deprivation (TSD) group and the control group. Significant differences were found in hit rate, false alarm, and d’ across the four consecutive days (baseline sleep (D2), TSD (D3), recovery sleep (D4), recovery sleep (D5)) in the TSD group. All three indexes of memory performances diminished significantly after TSD compared to baseline sleep (D2) (*p* < 0.05). However, task performances did not return to baseline levels (D2) after two consecutive nights of recovery sleep (D4 & D5) relative to TSD (D3) (*p* > 0.05). The differences in task performances between baseline sleep (D2) and first night of recovery sleep (D4; except hit rate) as well as second night of recovery sleep (D5) were both significant (*p* < 0.05). In the control group, no differences in task performances were found among corresponding days (*p* > 0.05). The baseline levels (D2) of all three indexes did not differ between groups (*p* > 0.05). *p < 0.05, **p < 0.01, ***p < 0.001.

We first compared behavioral performance on the episodic memory task before and after TSD to determine the effects of one night of acute TSD on memory function. As shown in Fig. 2, memory performance was significantly impaired after TSD (day 3) when compared to baseline (day 2), including significantly decreased hit rate (*p* < 0.05), increased false alarm (*p* < 0.01), and reduced d-prime (*p* < 0.001), while no differences were found in the memory performance between the corresponding day 2 and day 3 in the control subjects (all *p* > 0.05). These findings support our hypothesis (H1a) that one night of acute TSD would disrupt episodic memory function. We then compared memory performance at baseline (day 2) and after recovery sleep (day 5) to examine whether two nights of recovery sleep could fully restore episodic memory function. Contradicting with our initial hypothesis H2a, memory performance was still significantly impaired in hit rate (*p* < 0.05), false alarm (*p* < 0.05), and d-prime (*p* < 0.001), suggesting that participants may need more than two nights of recovery sleep to restore memory function after one night of sleep loss.

**Figure 3:**
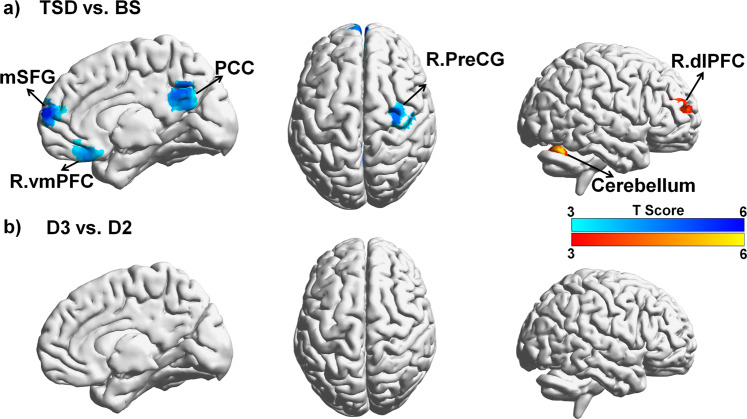
Functional connectivity analysis for both the total sleep deprivation (TSD) group and the control group, using bilateral hippocampus as the seed. (**a**) The contrast of TSD vs. BS showed significantly decreased connectivity between bilateral hippocampus and bilateral medial superior frontal gyrus (mSFG), posterior cingulated cortex (PCC), right ventromedial prefrontal cortex (R.vmPFC), as well as right precentral gyrus (R.PreCG), whereas increased connectivity between bilateral hippocampus and right dorsolateral frontal cortex (R.dlPFC) as well as bilateral cerebellum following TSD compared to following BS. Images were displayed at a threshold of uncorrected p < 0.001 with cluster size larger than 30 voxels. (**b**) In the control group, the contrast of D3 vs. D2 did not show any significant connectivity differences. BS: baseline sleep; TSD: total sleep deprivation.

For brain imaging data, we compared resting-state functional connectivity patterns before and after TSD to determine the effects of one night of acute TSD on hippocampal connectivity. As shown in Fig. 3a and listed in Table 1, hippocampal connectivity was significantly reduced in multiple DMN and prefrontal regions, including the posterior cingulate cortex (PCC), medial superior frontal gyrus (mSFG), right ventromedial prefrontal cortex (R.vmPFC), and right precentral gyrus (R.PreCG), and was significantly increased in bilateral cerebellum and right dorsolateral prefrontal cortex (R.dlPFC) after one night of TSD when compared to baseline condition. All of these brain regions survived whole brain family-wise error rate (FWE) corrected *p* < 0.01. These regions were defined as the regions of interest (ROIs) for further analyses to determine the restoration effects of two nights of recovery sleep on hippocampal connectivity. In contrast, no brain connectivity differences were found in the control group (see Fig. 3b). These findings support our hypothesis (H1b) that one night of acute TSD would significantly disrupt hippocampal connectivity.

**Table 1:** Brain regions showing significantly different hippocampal functional connectivity during total sleep deprivation (TSD) as compared to baseline (BS). All clusters survived whole brain FWE corrected p < 0.05. R. right, L. left.

Brain Regions	x, y, z	*p* value (FWE corrected)	Cluster Size	Peak T
**BS > TSD**				
Medial Superior Frontal Gyrus	3, 66, 15	<0.001	68	5.39
Posterior Cingulate Cortex	0, −54, 36	<0.001	145	4.83
R. Precentral Gyrus	33, −12, 66	<0.001	103	4.72
R. Ventromedial Prefrontal Cortex	6, 30, −24	<0.01	42	4.33
**TSD > BS**				
L. Cerebellum	−6, −72, −21	<0.001	114	5.68
R. Cerebellum	36,−63,−27	<0.01	56	5.31
R. Dorsolateral Prefrontal Cortex	36, 42, 18	<0.01	52	5.07

**Figure 4:**
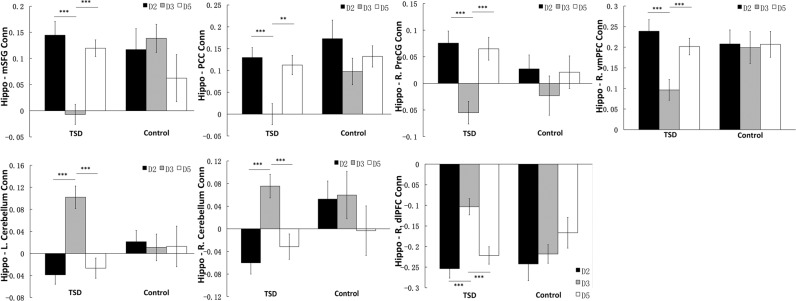
Region of interest (ROI) analysis for the total sleep deprivation (TSD) group and the control group. Functional connectivity between bilateral hippocampus and bilateral medial superior frontal gyrus (mSFG), posterior cingulated cortex (PCC), right precentral gyrus (R.PreCG), right ventromedial prefrontal cortex (R.vmPFC) decreased significantly after TSD (D3) relative to baseline sleep (D2) (*p* < 0.001), and increased significantly after recovery sleep (D5) relative to TSD (D3) (*p* < 0.001). The anti-correlation between bilateral hippocampus and bilateral cerebellum as well as right dorsolateral frontal cortex (R.dlPFC) reduced significantly after TSD (D3) relative to baseline sleep (D2) (*p* < 0.001), and returned back to baseline level (D2) after two nights of recovery sleep (D5) relative to TSD (D3) (*p* < 0.001). The differences in hippocampus-ROIs connectivity between baseline sleep (D2) and recovery sleep (D5) were not significant (*p* > 0.05). In the control group, no differences in connectivity were found among corresponding days (*p* > 0.05). The baseline levels (D2) of hippocampus-ROIs (except bilateral cerebellum) connectivity did not differ between groups (*p* > 0.05). Error bars represent standard errors of the mean. *p < 0.05, **p < 0.01, ***p < 0.001.

The results from the ROI analyses are shown in Fig. 4. When comparing hippocampal connectivity at baseline (day 2) and after recovery sleep (day 5), no differences were observed in the TSD group (all *p* > 0.05). These findings support our hypothesis (H2b) and suggest that two nights of recovery sleep would restore hippocampal connectivity to baseline levels after one night of sleep loss. Moreover, the ROI analyses also demonstrated that the increased connectivity in the R.dlPFC from the whole-brain analysis was due to reduced negative connectivity (anti-correlations) between the hippocampus and this region (*p* < 0.001), and the increased connectivity in the cerebellum was due to reversed connectivity (from negative to positive correlations) to the hippocampus during TSD (day 3) as compared to baseline (day 2). No connectivity differences were found among any scans from the three corresponding days in the control subjects (all *p* > 0.05).

**Figure 5:**
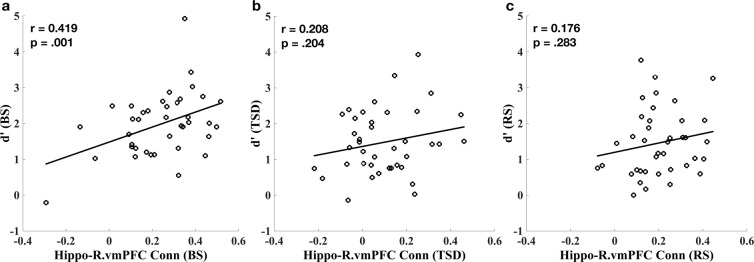
The scatter plots showed the correlation between hippocampal connectivity and episodic memory performance (d’) after baseline sleep (BS, **a**), during total sleep deprivation (TSD, **b**), and following recovery sleep (RS, **c**).

We also examined the relationships between hippocampal connectivity and episodic memory performance (d-prime) separately during each scan day (day 2, day 3, and day 5, see Fig. 5) using correlation analyses and non-parametric permutation tests. A significant positive correlation was observed between resting-state hippocampal connectivity and memory performance (d-prime) in the R.vmPFC (Fig. 5a, Pearson r = 0.419, *p* = 0.001; Spearman rho = 0.336, *p* = 0.036; permutation test *p* = 0.003). However, this correlation was disrupted during TSD (Fig. 5b, Pearson r = 0.208, *p* = 0.20; Spearman rho = 0.144, *p* = 0.38; permutation test *p* = 0.11) and following recovery sleep (Fig. 5c, Pearson r = 0.176, *p* = 0.28; Spearman rho = 0.126, *p* = 0.26; permutation test *p* = 0.13). Although the comparisons of correlation coefficients did not reach significant differences between conditions, these findings suggest that TSD impaired the associations between hippocampal connectivity and episodic memory performance and such impairments might not be fully restored after two nights of recovery sleep.

## Discussion

To our knowledge, this study demonstrated for the first time both the detrimental and restoring changes in resting-state hippocampal connectivity and episodic memory after one night of acute total sleep deprivation and following two consecutive nights of recovery sleep. As expected, we found significantly impaired connectivity between hippocampus and multiple brain regions after TSD, which is in line with previous memory and sleep deprivation studies^14,15,20,22,23,38,47–49^. However, we also found that two nights of recovery sleep following TSD did not restore episodic memory performance to baseline, even though hippocampal connectivity was restored to baseline levels. These findings only partly support our hypothesis concerning the restoring effects of recovery sleep but are nevertheless consistent with several previous studies showing insufficient restoration of cognitive performance and/or brain function after one or two nights of recovery sleep following sleep loss^1,3,41–45^. In addition, the associations between hippocampal connectivity and episodic memory performance were also disrupted during TSD and did not fully restore after two nights of recovery sleep, suggesting that intact hippocampal functional connectivity may not be sufficient to support normal episodic memory function after two nights of recovery from sleep deprivation.

Numerous previous studies have undoubtedly demonstrated the impacts of sleep loss on multiple neurobehavioral domains, particularly attention, learning, and memory^7,8,10,11,21,41,50,51^. Impaired neurobehavioral performance includes slowed response times, concentration lapses, increased errors of commission and omission, and mitigated learning ability and memory consolidation. For example, using a picture recognition memory task, Yoo and colleagues^15^ found that a single night of total sleep deprivation produced significant deficits in hippocampus-dependent episodic memory encoding and subsequent memory retention, while Sterpenich and colleagues^14^ showed that one night of total sleep deprivation impaired recollection of positive and neutral pictures (but not negative pictures). Moreover, Van Der Werf and colleagues^27^ reported that even a mild sleep disruption was sufficient to affect hippocampal-dependent memory encoding and performance. Using a free recall test of word lists, de Almeida and colleagues^38^ reported that both episodic memory and short-term memory were significantly spared after two nights of sleep deprivation. In our study, we found that one night of TSD significantly reduced hit rate, increased false alarm, and reduced d-prime, which are consistent with previous studies and further support the key role of sleep in hippocampus-dependent episodic memory function. Memory performance did not show any significant changes across three scan days in the control protocol without sleep deprivation, suggesting that hindered memory performance was specific to sleep loss and not due to other aspects of the experimental protocol that the control participants also experienced.

TSD altered functional connectivity between the hippocampus and multiple DMN and prefrontal regions, including the PCC, vmPFC, and mSFG. These findings replicate previous studies showing that TSD impaired hippocampal connectivity at rest and/or during memory tasks^14,15,20,22,23^. Both the PCC and vmPFC are the core nodes of DMN, which is thought to be involved in autobiographical and episodic memory processing^52–54^. Enhanced connectivity between the hippocampus and DMN has been linked to successful memory formation in normal individuals^55,56^ while reduced connectivity has been observed in patients with episodic memory deficits^57,58^. However, we did not find increased hippocampal connectivity to subcortical regions after TSD as reported in several previous studies^15,22^. The discrepancy may be due to differences in the experimental design, study protocol, characteristics of participants, as well as data acquisition parameters and analysis pipelines. For example, the study by Yoo *et al*.^15^ employed a between-subject design and compared hippocampal connectivity between a cohort of sleep-deprived subjects and another independent cohort of non-sleep-deprived controls during the performance of a memory encoding task. In contrast, both the current study and the study by Chengyang *et al*.^22^ employed a within-subject design and compared resting-state hippocampal connectivity in the same cohort of healthy adults at rested wakefulness and after one night of sleep deprivation. The Chengyang *et al*. study^22^ included two scans from 20 healthy young male adults (age range 18–24 years) while the current study included three scans from 70 male and female healthy adults with a much larger age range (21–50 years). In addition, all subjects in the current study were required to stay in the laboratory across 5 days and 4 nights and carefully monitored for their activities and food intake, while subjects in previous studies were monitored in the laboratory during the TSD period. However, it remains unknown how the laboratory environment and specific activities would affect hippocampal connectivity and memory function.

We also found that TSD significantly increased hippocampal connectivity to the right dlPFC, a key node of the task-positive dorsal attention network (DAN). However, ROI analyses indicated that such increased connectivity was actually due to reduced anti-correlation between the dlPFC and the hippocampus. Since the hippocampus is a core region of the DMN^54,59,60^, this finding is consistent with the literature demonstrating reduced anti-correlations between the DMN and DAN after sleep loss^20,61–65^. During rested wakefulness after normal sleep, the task-negative DMN is usually activated whereas the task-positive DAN is suppressed at rest in the absence of task requirements, suggesting a functional decoupling between these two networks^66,67^. The reduction of anti-correlation during TSD suggests that task-negative and task-positive networks may fail to remain functionally distinct from each other after sleep loss, which may lead to an altered allocation of cognitive resources to brain networks and poor modulation of attention processes in response to shifting cognitive demands^21,68,69^.

One night of TSD not only impaired resting-state hippocampal connectivity, but also disrupted the associations between hippocampal connectivity and episodic memory performance. Specifically, resting-state hippocampal connectivity to prefrontal regions (R.vmPFC) correlated with episodic memory performance following baseline sleep, but not during sleep deprivation or after recovery sleep. These findings are consistent with the view that hippocampus-prefrontal communications are important for optimal redistribution of temporal memory traces to more permanent cortical storage, and that disruptions of such communications may compromise the cognitive capacity for committing new experiences to memory^15^.

Consistent with our hypothesis, we found that disrupted hippocampal connectivity after TSD returned to baseline levels after two consecutive nights (20 hours of time-in-bed) of recovery sleep. However, we did not observe full restoration of memory performance and its associations with hippocampal connectivity to baseline levels after two consecutive nights of recovery sleep. These findings on memory restoration failure appear to contradict several prior studies where it was suggested that one night of recovery sleep restored cognitive performance^34–36,38,39,70^ to baseline levels. Specifically, a previous study reported that a 90-min recovery nap restored hippocampus-dependent learning ability and memory function in a mnemonic similarity task during the day of acute total sleep deprivation^47^. However, the failure of memory restoration after two nights of recovery sleep is in line with many other previous studies where it was indicated that more than one night of recovery sleep was needed to fully restore cognitive performance^1,3,37,41,43–46^ and brain metabolic changes^42^ after total or chronic sleep loss. For example, Dinges and colleagues^41^ reported that one night of recovery sleep was not sufficient to reverse cognitive deficits resulting from chronic sleep restriction. Similarly, Belenky and colleagues^1^ showed that three nights of recovery sleep were not able to fully restore performance in the psychomotor vigilance task (PVT) after seven nights of chronic sleep restriction. Pejovic and colleagues^44^ showed that PVT performance deficits persisted after two nights of recovery sleep following six nights of chronic sleep restriction (6-h). Lo and colleagues^45^ also reported that sustained attention performance did not return to baseline levels after two nights of recovery sleep following seven nights of chronic sleep restriction (5-h). Taken together, our findings provide further evidence supporting that two nights of recovery sleep are insufficient to fully restore cognitive and brain function from one night of TSD.

There are several limitations in our study. First, this study only involves overnight total sleep deprivation, thus we could not determine the specific contribution of different types of sleep, such as slow wave sleep (SWS) or rapid eye movement (REM) sleep to the detrimental effects of sleep loss on hippocampal connectivity and episodic memory. Moreover, we were not able to examine the sleep stage changes during the two night of recovery sleep. However, the dual-process hypothesis suggests that SWS is more important for declarative memory (including episodic memory and semantic memory) whereas REM sleep is essential for procedural memory^71–75^. Increased functional coupling between the hippocampus and neocortical regions has been found in stage-2 sleep^76^, while decreased coupling between the hippocampus and PCC has been found during SWS compared with the waking state^77^. Furthermore, increases in hippocampal connectivity correlate with the amount of prior SWS^78^. These findings suggest that different types of memories may occur during different sleep stages. Future studies should replicate the current findings and determine the potential different roles of stage-2 sleep, SWS, and REM sleep in maintaining and restoring brain network integrity and memory function.

Second, although both acute TSD and chronic sleep restriction can induce similar impairments in cognitive performance, some studies suggest that the recovery following acute TSD may be faster than that following chronic sleep restriction^3,34,70^. Therefore, acute TSD and chronic sleep restriction might have somewhat different physiological processes^1,79,80^. The present study focuses on the effects of acute TSD and their restoration, thus our findings cannot be generalized to the effects of chronic partial sleep restriction on brain function and its associations with memory performance. Future studies are needed to determine whether acute total sleep deprivation and chronic partial sleep restriction and their recovery would share the same or separable neural mechanisms, and whether neural recovery would occur across the same time period or over a different temporal duration following different forms and doses of sleep deprivation^21^.

Third, we only used fMRI to measure resting-state hippocampal connectivity and did not image memory task in the scanner at the same time. Previous task-related fMRI studies have reported significant effects of sleep deprivation and recovery sleep on memory performance and hippocampal function^14,15,27,81^. However, because sleep deprivation simultaneously impairs brain function and memory performance, it is difficult for task-related fMRI studies to dissociate the effects of sleep loss on brain function *per se* from the effects of sleep loss on task performance that subsequently affects brain function. Moreover, the use of resting-state fMRI may eliminate confounds of differences in task performance, effort, practice, task strategy^82,83^, and reduce the potential effects of fatigue on brain connectivity. Of note, sleep deprived participants may easily become fatigued when performing tasks, therefore, the use of resting-state fMRI outperforms the use of task-based fMRI with respect to sleep deprivation studies. Nonetheless, future studies may need to combine both resting-state and task-related fMRI to more comprehensively understand the impairing effects of sleep deprivation and the restoring effects of recovery sleep on brain function and memory performance.

Finally, the final analyses only included 39 subjects with an age range between 21 and 50 years. Although this sample size is not small in the sleep deprivation literature, we were not able to find significant differences in the correlation coefficients between memory performance (d-prime) and the hippocampal connectivity across baseline, TSD, and recovery sleep conditions. Thus, our results of the disrupted relationships between memory performance and hippocampal connectivity after TSD and recovery sleep need to be regarded as preliminary and future studies with a larger sample size are needed to validate our findings. In addition, because memory degradation is highly prevalent in elderly individuals, future studies are necessary to examine how sleep loss and recovery sleep impact memory function in this vulnerable population. However, our findings may have important implications for understanding memory dysfunction in aging and neurodegenerative diseases. For example, hippocampal connectivity measured by resting-state fMRI may offer a noninvasive approach for monitoring the effects of sleep loss on the episodic memory system in the human brain. Disturbed hippocampal connectivity has been consistently observed in mild cognitive impairment and Alzheimer’s Disease (AD), for which episodic memory degradation is a hallmark symptom^57,58^. Both animal and human studies have demonstrated that sleep loss exacerbates amyloid-beta (Aβ) plaque aggregation in the brain, a fundamental process leading to AD^84–87^. Moreover, a very recent study suggests that quantitative and qualitative features of human sleep may represent non-invasive biomarkers of AD pathology^88^. Since reduced sleep duration and increased sleep disturbances are concomitant symptoms of aging and AD^89–92^, disruption in hippocampal connectivity and its associations with memory performance may provide a plausible mechanism for the deleterious effects of sleep loss in this population.

In summary, here we used resting-state functional magnetic resonance imaging in a well controlled in-laboratory sleep deprivation protocol and demonstrated both the impairing effects of one night of acute total sleep deprivation and the restoring effects of two consecutive nights of recovery sleep on hippocampal connectivity and episodic memory performance. We found that sleep deprivation significantly impaired hippocampal connectivity to multiple prefrontal and default mode network regions, reduced episodic memory performance, and disrupted the associations between them. Although hippocampal connectivity was fully restored to baseline levels after two nights of recovery sleep, episodic memory performance and its associations with hippocampal connectivity did not return to baseline. These findings support the critical role of sleep in maintaining the integrity of hippocampal circuits and suggest that people may need more than two nights of recovery sleep to fully restore both memory performance and their associations with brain function after one night of total sleep loss. Optimization of sleep time may represent a potential effective intervention for the deleterious effects of sleep loss on memory and hippocampal function, with therapeutic opportunities for aging and AD.

## Methods

### Participants

A total of 70 healthy adults (age range 21–50 years, 41 males) participated in a 5-day and 4-night in-laboratory controlled sleep deprivation experiment^93,94^, including 54 adults in the experimental group with one night of TSD (Fig. 1a) and 16 adults in the control group without sleep loss (Fig. 1b). Fifteen participants in the TSD group and one participant in the control group were excluded due to head motion, MR hardware problem, falling asleep during the scans, and/or missing behavioral data. Thus, fifty-four participants (age range 21–50 yrs, 28 males) were included in the present study, including 39 in the TSD group (mean age = 33.5 ± 8.8 yrs, 22 males) and 15 in the control group (mean age = 34.5 ± 9.0 yrs, 7 males). The sleep-wake schedule of each participant was assessed by at least one week actigraphy, sleep-wake diaries, sleep and circadian rhythm questionnaires, and a night of laboratory polysomnography and oximetry measurements during the weeks preceding the study. All participants had a regular sleep-wake schedule of nocturnal sleep duration between 6.5 and 8.5 hours, regular bedtime between 2200–0000 h and wake time of 0600–0900 h, and no evidence of habitual napping or sleep disorders. Participants could not have participated in trans-meridian travel or shift work, nor had irregular sleep-wake routines for 60 days prior to the in-lab study. Caffeine, alcohol, tobacco, medications (except 4 subjects took oral contraceptives and one subject took Mirena for birth control) or other psychoactive substances were prohibited from the week prior to and during the in-laboratory study. All participants were right-handed, not current smokers, and had no history of chronic physical or mental illness, as established by interviews, questionnaires, physical examinations, and blood and urine tests. This study was approved by the Institutional Review Board of the University of Pennsylvania and was carried out in accordance with the Declaration of Helsinki. All participants provided written informed consent before enrollment and were compensated for participating in the study.

### In-laboratory study protocol

The in-laboratory study lasted 5 consecutive days and 4 nights (see Fig. 1a for the study protocol). All participants stayed in a laboratory room of the Clinical Translational Research Center at the Hospital of the University of Pennsylvania. Participants arrived at the laboratory on the afternoon of day 1 and were provided 9–10 hours time-in-bed (TIB) sleep opportunity for baseline sleep on night 1. The first fMRI scan session took place on the morning of day 2 (0700–1000 hours). Participants were then randomized to either a TSD or a control condition. On night 2, participants in the SD group were kept awake and then underwent the second fMRI scan on the morning of day 3 (0700–1000 hours). On nights 3 and 4, these participants received 12 and 8 hours recovery sleep, respectively. We used two consecutive nights of recovery sleep because previous studies have suggested that neurobehavioral and brain activity deficits after sleep loss may not be fully recovered after one night of sleep^3,42^. The third fMRI scan took place on the morning of day 5 (0700–1000 hours). Participants in the control group (Fig. 1b) also had the same three fMRI scans on the mornings of days 2, 3, and 5, but they were provided 8 hours TIB sleep on nights 2 to 4. Because previous studies have suggested that time-of-day may modulate brain activity and memory performance^95–104^, the time of fMRI scans and cognitive tests were fixed for all subjects and kept constant across the whole protocol to minimize the potential confounding time-of-day effects.

Throughout the 5-day protocol, participants were continuously behaviorally monitored by trained staff in a semi-isolated living area. They were provided meals at regular, pre-specified hours, as well as snacks within reasonable limits. When not testing during the study, they were permitted to engage in non-strenuous activities such as reading and watching television. They were not allowed to do any physically demanding tasks, work on laptops, or leave the study environment.

During the afternoons (1400–1800 hours) of days 2 to 5, all participants completed a scene encoding and recognition task to measure episodic memory function after SD. For the episodic memory task, participants were asked to view 90 or 150 color pictures during the encoding session and told to remember them for later recognition testing. Each day participants learned a different set of picture stimuli consisting of scenes, landscapes, objects, and non-famous people, which were pseudo-randomly selected from the International Affective Pictures System (IAPS)^105^ and online, The number of pictures was kept the same across different days. Pictures with extreme emotional valence were excluded. The pictures were matched in terms of overall visual complexity, brightness, contrast, and emotional valence. Each picture was displayed on the computer screen for 1 second with 0.5 second inter-stimulus interval. A few minutes after the encoding task, participants performed the recognition tests, in which they viewed 30 pictures from the encoding session mixed with 60 new pictures as foils. Participants were required to make a forced-choice response as to whether they remembered the picture from the encoding session (‘old’) or believed they had not viewed the picture before (‘new’). There were four types of responses: (i) old pictures correctly recognized as old (‘hits’); (ii) new stimuli correctly recognized as new (‘correct rejections’); (iii) old pictures incorrectly judged to be new (‘misses’); and (iv) new pictures incorrectly judged to be old (‘false alarms’). Task performance was calculated on the basis of hit rate [hits/(hits + misses)], false alarm (FA) rate [false alarms/(false alarms + correct rejections)], and signal detection accuracy d-prime (d’). According to the signal detection theory, d-prime is calculated as the normalized distance between the probability distributions of signal and noise and noise alone^106^. Greater d-prime indicates participants have better performance to discriminate target from nontarget therefore a better memory in the task. Differences in episodic memory performance across the four consecutive days (days 2–5) were analyzed using repeated measures one-way analyses of variance (ANOVA).

### Imaging data acquisition and analysis

All MRI scans were performed using a 3 T Siemens Trio system (Siemens Medical Systems, Erlangen, Germany). A multiband gradient-echo EPI sequence was used for resting-state BOLD fMRI data acquisition with the following parameters: TR = 2 s, TE = 24 ms, FOV = 220 × 220 mm, matrix = 64 × 64 × 36, slices thickness = 4 mm, 36 interleaved slices without gap. A total of 210 images were acquired from about 7 min scanning for each participant. Participants were instructed to remain still in the scanner, keep their eyes open and look at a cross fixation on the center of a screen. An eye-tracking camera was used to monitor participants’ eyes to confirm that they did not fall asleep during the scan. After the functional scans, high-resolution (1 × 1 × 1 mm^3^) T1-weighted anatomic images were obtained using a standard 3D MPRAGE sequence for structural reference.

Image data processing and analyses were carried out with the Statistical Parametric Mapping software (SPM 12, Wellcome Department of Cognitive Neurology, UK) and the REST 2.0 toolbox (http://resting-fmri.sourceforge.net/), implemented in Matlab 14 (Math Works, Natick, MA). The 3 translational/rotational motion parameters and the framewise displacement (FD) were calculated. To control for the effect of head motion, the threshold of head motion parameters were set as 2 for translational/rotational motion and 0.5 for mean FD. Data from subjects whose head motion parameters were over the threshold were discarded. After head motion correction, scrubbing, and co-registration, functional images were smoothed using an isotropic Gaussian kernel with a full-width at half-maximum (FWHM) of 4 mm and then normalized to the standard Montreal Neurological Institute (MNI) space. Linear trends were also removed. All functional volumes were finally band pass filtered at 0.01–0.08 Hz to reduce low-frequency drift and physiological high-frequency respiratory and cardiac noise. Nuisance covariates including six head motion parameters, global mean signal, white matter signal and CSF signal were regressed out before the seed-based functional connectivity (FC) analysis^107^.

The hippocampal seed was defined as the bilateral hippocampus from an automated anatomical labeling region of interest library^108^. The Pearson’s correlation coefficients between the mean BOLD fMRI signal time series of the seed region and all other voxels within the brain were calculated, generating a correlation map for each participant. Fisher’s r-to-z transformation {z = 0.5 Ln [(1 + r)/(1 − r)]} was then applied on these correlation maps to improve the normality of the correlation coefficients. A whole-brain voxel-wise group-level paired t-test was performed on these z-transformed correlation maps to compare resting-state brain connectivity patterns before and after TSD and determine the effects of one night of acute TSD on hippocampal connectivity. The threshold was defined as whole brain family-wise error (FWE) corrected *p* < 0.05. The regions showing significant changes in connectivity to hippocampus were then defined as the regions of interest (ROIs) to determine the restoration effects of two nights of recovery sleep on hippocampal connectivity. Spearman and Pearson correlation analyses, as well as permutation tests were performed to examine the relationships between hippocampal connectivity and episodic memory performance (d-prime) separately during each scan day using SPSS Statistics for Windows (Version 18.0, IBM, Chicago, IL). Specifically, we used a permutation testing procedure to test the significance of the Pearson correlation: (i) the observed Pearson correlation was computed between the hippocampus-R.vmPFC connectivity and the d-prime; (ii) the values of d-prime were permuted relative to the connectivity values; (iii) Pearson correlation was then re-computed after each permutation; (iv) (i) and (ii) were repeated 10,000 times to build a null distribution of Pearson correlations for comparison with the observed Pearson correlation. Moreover, we used a correlation comparison method suggested by Diedenhofen and Musch^109^ to evaluate whether correlation coefficients between memory performance (d-prime) and the hippocampal connectivity were statistically different across baseline, TSD, and recovery sleep conditions.

## References

[1] Belenky G (2003). Patterns of performance degradation and restoration during sleep restriction and subsequent recovery: a sleep dose-response study. J. Sleep Res..

[2] Van Dongen HPA, Maislin G, Mullington JM, Dinges DF (2003). The Cumulative Cost of Additional Wakefulness: Dose-Response Effects on Neurobehavioral Functions and Sleep Physiology From Chronic Sleep Restriction and Total Sleep Deprivation. Sleep.

[3] Banks S, Van Dongen HPA, Maislin G, Dinges DF (2010). Neurobehavioral dynamics following chronic sleep restriction: Dose-response effects of one night for recovery. Sleep.

[4] Ford ES, Cunningham TJ, Croft JB (2015). Trends in Self-Reported Sleep Duration among US Adults from 1985 to 2012. Sleep.

[5] Watson NF (2015). Recommended Amount of Sleep for a Healthy Adult: A Joint Consensus Statement of the American Academy of Sleep Medicine and Sleep Research Society. Sleep.

[6] Banks S, Dinges DF (2007). Behavioral and Physiological Consequences of Sleep Restriction. J. Clin. Sleep Med..

[7] Lim J, Dinges DF (2008). Sleep deprivation and vigilant attention. Ann. N. Y. Acad. Sci..

[8] Goel N, Rao H, Durmer JS, Dinges DF (2009). Neurocognitive Consequences of Sleep Deprivation. Semin Neurol..

[9] Spaeth AM, Dinges DF, Goel N (2013). Effects of Experimental Sleep Restriction on Weight Gain, Caloric Intake, and Meal Timing in Healthy Adults. Sleep.

[10] Walker MP, Stickgold R (2004). Sleep-dependent learning and memory consolidation. Neuron.

[11] Diekelmann S, Born J (2010). The memory function of sleep. Nat. Rev. Neurosci..

[12] Inostroza M, Born J (2013). Sleep for Preserving and Transforming Episodic Memory. Annu. Rev. Neurosci..

[13] Fernandes C (2015). Detrimental role of prolonged sleep deprivation on adult neurogenesis. Front. Cell. Neurosci..

[14] Sterpenich V (2007). Sleep-related hippocampo-cortical interplay during emotional memory recollection. PLoS Biol..

[15] Yoo SS, Hu PT, Gujar N, Jolesz FA, Walker MP (2007). A deficit in the ability to form new human memories without sleep. Nat. Neurosci..

[16] Havekes R, Vecsey CG, Abel T (2012). The impact of sleep deprivation on neuronal and glial signaling pathways important for memory and synaptic plasticity. Cell. Signal..

[17] Havekes R (2016). Sleep deprivation causes memory deficits by negatively impacting neuronal connectivity in hippocampal area CA1. Elife.

[18] Abel T, Havekes R, Saletin JM, Walker MP (2013). Sleep, plasticity and memory from molecules to whole-brain networks. Curr. Biol..

[19] Menz MM (2013). The role of sleep and sleep deprivation in consolidating fear memories. Neuroimage.

[20] Yeo BTT, Tandi J, Chee MWL (2015). Functional connectivity during rested wakefulness predicts vulnerability to sleep deprivation. Neuroimage.

[21] Krause AJ (2017). The sleep-deprived human brain. Nat. Rev. Neurosci..

[22] Chengyang L (2017). Short-term memory deficits correlate with hippocampal-thalamic functional connectivity alterations following acute sleep restriction. Brain Imaging Behav..

[23] Zhao R (2019). Disrupted Resting-State Functional Connectivity in Hippocampal Subregions After Sleep Deprivation. Neuroscience.

[24] Sirota A, Csicsvari J, Buhl D, Buzsa´ki G (2003). Communication between neocortex and hippocampus during sleep in rodents. Proc. Natl. Acad. Sci..

[25] Ji D, Wilson MA (2007). Coordinated memory replay in the visual cortex and hippocampus during sleep. Nat. Neurosci..

[26] Logothetis NK (2012). Hippocampal-cortical interaction during periods of subcortical silence. Nature.

[27] Van Der Werf YD (2009). Sleep benefits subsequent hippocampal functioning. Nat. Neurosci..

[28] Marshall L, Born J (2007). The contribution of sleep to hippocampus-dependent memory consolidation. Trends Cogn. Sci..

[29] Schlichting ML, Preston AR (2014). Memory reactivation during rest supports upcoming learning of related content. Proc. Natl. Acad. Sci..

[30] Voets NL (2014). Aberrant Functional Connectivity in Dissociable Hippocampal Networks Is Associated with Deficits in Memory. J. Neurosci..

[31] Cooper RA (2017). Reduced Hippocampal Functional Connectivity During Episodic Memory Retrieval in Autism. Cereb. cortex.

[32] Tompary A, Davachi L (2017). Consolidation Promotes the Emergence of Representational Overlap in the Hippocampus and Medial Prefrontal Cortex. Neuron.

[33] Vecsey CG (2009). Sleep deprivation impairs cAMP signalling in the hippocampus. Nature.

[34] Drummond SPA, Paulus MP, Tapert SF (2006). Effects of two nights sleep deprivation and two nights recovery sleep on response inhibition. J. Sleep Res..

[35] Jay SM (2007). The characteristics of recovery sleep when recovery opportunity is restricted. Sleep.

[36] Mander BA (2010). EEG measures index neural and cognitive recovery from sleep deprivation. J. Neurosci..

[37] Tucker AM, Whitney P, Belenky G, Hinson JM, Van Dongen HPA (2010). Effects of Sleep Deprivation on Dissociated Components of Executive Functioning. Sleep.

[38] de Almeida VZG (2012). Free Recall of Word Lists under Total Sleep Deprivation and after Recovery Sleep. Sleep.

[39] Philip P (2012). Acute Versus Chronic Partial Sleep Deprivation in Middle-Aged People: Differential Effect on Performance and Sleepiness. Sleep.

[40] Elmenhorst D (2017). Recovery sleep after extended wakefulness restores elevated A1 adenosine receptor availability in the human brain. Proc. Natl. Acad. Sci..

[41] Dinges DF (1997). Cumulative Sleepiness, Mood Disturbance, and Psychomotor Vigilance Performance Decrements During a Week of Sleep Restricted to 4-5 Hours per Night. Sleep.

[42] Wu JC (2006). Frontal Lobe Metabolic Decreases with Sleep Deprivation not Totally Reversed by Recovery Sleep. Neuropsychopharmacology.

[43] Ikegami K (2009). Recovery of cognitive performance and fatigue after one night of sleep deprivation. J. Occup. Health.

[44] Pejovic S (2013). Effects of recovery sleep after one work week of mild sleep restriction on interleukin-6 and cortisol secretion and daytime sleepiness and performance. Am. J. Physiol. Metab..

[45] Lo JC, Ong JL, Leong RLF, Gooley JJ, Chee MWL (2016). Cognitive Performance, Sleepiness, and Mood in Partially Sleep Deprived Adolescents: The Need for Sleep Study. Sleep.

[46] Boardman JM (2018). The ability to self-monitor cognitive performance during 60 h total sleep deprivation and following 2 nights recovery sleep. J. Sleep Res..

[47] Saletin JM (2016). Human Hippocampal Structure: A Novel Biomarker Predicting Mnemonic Vulnerability to, and Recovery from, Sleep Deprivation. J. Neurosci..

[48] Hu P, Stylos-allan M, Walker MP (2006). Sleep faciliates consolidation of emotional declarative memory. Psychol. Sci..

[49] Chuah LYM (2009). Donepezil Improves Episodic Memory in Young Individuals Vulnerable to the Effects of Sleep Deprivation. Sleep.

[50] Drummond SPA (2000). Altered brain response to verbal learning following sleep deprivation. Nature.

[51] Killgore, W. D. S. Effects of sleep deprivation on cognition. Progress in Brain Research 185, (Elsevier B.V., 2010). 10.1016/B978-0-444-53702-7.00007-5 21075236

[52] Gusnard DA, Akbudak E, Shulman GL, Raichle ME (2001). Medial prefrontal cortex and self-referential mental activity: Relation to a default mode of brain function. Proc. Natl. Acad. Sci..

[53] Vincent JL (2006). Coherent Spontaneous Activity Identifies a Hippocampal-Parietal Memory Network. J Neurophysiol.

[54] Buckner RL, Andrews-Hanna JR, Schacter DL (2008). The brain’s default network: Anatomy, function, and relevance to disease. Ann. N. Y. Acad. Sci..

[55] Ranganath C, Heller A, Cohen MX, Brozinsky CJ, Rissman J (2005). Functional connectivity with the hippocampus during successful memory formation. Hippocampus.

[56] Schott BH (2013). The relationship between level of processing and hippocampal-cortical functional connectivity during episodic memory formation in humans. Hum. Brain Mapp..

[57] Greicius MD, Srivastava G, Reiss AL, Menon V (2004). Default-mode network activity distinguishes Alzheimer’s disease from healthy aging: evidence from functional MRI. Proc. Natl. Acad. Sci..

[58] Sperling RA (2010). Functional alterations in memory networks in early alzheimer’s disease. NeuroMolecular Med..

[59] Raichle ME (2001). A default mode of brain function. Proc. Natl. Acad. Sci..

[60] Vincent JL, Kahn I, Snyder AZ, Raichle ME, Buckner RL (2008). Evidence for a Frontoparietal Control System Revealed by Intrinsic Functional Connectivity. J. Neurophysiol..

[61] Sämann PG (2010). Increased sleep pressure reduces resting state functional connectivity. Magn. Reson. Mater. Physics, Biol. Med..

[62] Havas JAD, Parimal S, Soon CS, Chee MWL (2012). Sleep deprivation reduces default mode network connectivity and anti-correlation during rest and task performance. Neuroimage.

[63] Bosch OG (2013). Sleep deprivation increases dorsal nexus connectivity to the dorsolateral prefrontal cortex in humans. Proc. Natl. Acad. Sci..

[64] Lei Y (2015). Large-Scale Brain Network Coupling Predicts Total Sleep Deprivation Effects on Cognitive Capacity. PLoS One.

[65] Nilsonne G (2017). Intrinsic brain connectivity after partial sleep deprivation in young and older adults: Results from the Stockholm Sleepy Brain study. Sci. Rep..

[66] Fox MD (2005). The human brain is intrinsically organized into dynamic, anticorrelated functional networks. Proc. Natl. Acad. Sci..

[67] Fox MD, Raichle ME (2007). Spontaneous fluctuations in brain activity observed with functional magnetic resonance imaging. Nat. Rev. Neurosci..

[68] Clapp WC, Rubens MT, Sabharwal J, Gazzaley A (2011). Deficit in switching between functional brain networks underlies the impact of multitasking on working memory in older adults. Proc. Natl. Acad. Sci..

[69] Turner GR, Spreng RN (2012). Executive functions and neurocognitive aging: Dissociable patterns of brain activity. Neurobiol. Aging.

[70] Lamond N (2007). The dynamics of neurobehavioural recovery following sleep loss. J. Sleep Res..

[71] Smith C (1995). Sleep states and memory processes. Behav. Brain Res..

[72] Plihal W, Born J (1997). Effects of early and late nocturnal sleep on declarative and procedural memory. J. Cogn. Neurosci..

[73] Ficca G, Salzarulo P (2004). What in sleep is for memory. Sleep Med..

[74] Stickgold R (2005). Sleep-dependent memory consolidation. Nature.

[75] Rasch B, Born J (2013). About Sleep’s Role in Memory. Physiol. Rev..

[76] Andrade KC (2011). Sleep Spindles and Hippocampal Functional Connectivity in Human NREM Sleep. J. Neurosci..

[77] Sämann PG (2011). Development of the brain’s default mode network from wakefulness to slow wave sleep. Cereb. Cortex.

[78] Mander BA (2013). Prefrontal atrophy, disrupted NREM slow waves and impaired hippocampal-dependent memory in aging. Nat. Neurosci..

[79] Alhola P, Polo-Kantola P (2007). Sleep deprivation: Impact on cognitive performance. Neuropsychiatr. Dis. Treat..

[80] Basner M, Rao H, Goel N, Dinges DF (2013). Sleep deprivation and neurobehavioral dynamics. Curr. Opin. Neurobiol..

[81] Gujar N, Yoo SS, Hu P, Walker MP (2010). The unrested resting brain: Sleep deprivation alters activity within the default-mode network. J. Cogn. Neurosci..

[82] Fox MD, Greicius M (2010). Clinical applications of resting state functional connectivity. Front. Syst. Neurosci..

[83] Sanchez Panchuelo R.M., Stephenson M.C., Francis S.T., Morris P.G. (2014). Neural brain activation imaging. Biomedical Imaging.

[84] Kang J-E (2009). Amyloid-β Dynamics Are Regulated by Orexin and the Sleep-Wake Cycle. Science (80-.)..

[85] Jack CR (2013). Tracking pathophysiological processes in Alzheimer’s disease: An updated hypothetical model of dynamic biomarkers. Lancet Neurol..

[86] Gouras GK, Olsson TT, Hansson O (2015). β-amyloid Peptides and Amyloid Plaques in Alzheimer’s Disease. Neurotherapeutics.

[87] Shokri-Kojori Ehsan, Wang Gene-Jack, Wiers Corinde E., Demiral Sukru B., Guo Min, Kim Sung Won, Lindgren Elsa, Ramirez Veronica, Zehra Amna, Freeman Clara, Miller Gregg, Manza Peter, Srivastava Tansha, De Santi Susan, Tomasi Dardo, Benveniste Helene, Volkow Nora D. (2018). β-Amyloid accumulation in the human brain after one night of sleep deprivation. Proceedings of the National Academy of Sciences.

[88] Winer JR (2019). Sleep as a potential biomarker of Tau and β-amyloid burdern in the human brain. J. Neurosci..

[89] Vitiello MV, Borson S (2001). Sleep disturbances in patients with alzheimer’s disease: Epidemiology, pathophysiology and treatment. CNS Drugs.

[90] Gagnon J-F, Petit D, Latreille V, Montplaisir J (2008). Neurobiology of Sleep Disturbances in Neurodegenerative Disorders. Curr. Pharm. Des..

[91] Pace-Schott, E. F. & Spencer, R. M. C. Age-related changes in the cognitive function of sleep. Progress in Brain Research 191, (Elsevier B.V., 2011). 10.1016/B978-0-444-53752-2.00012-6 21741545

[92] Harand C (2012). How aging affects sleep-dependent memory consolidation?. Front. Neurol..

[93] Fang Z (2015). Altered salience network connectivity predicts macronutrient intake after sleep deprivation. Sci. Rep..

[94] Yang FN (2018). Sleep deprivation enhances inter-stimulus interval effect on vigilant attention performance. Sleep.

[95] Davies JA, Navaratnam V, Redfern PH (1973). A 24-hour rhythm in passive-avoidance behaviour in rats. Psychopharmacologia.

[96] Folkard S (1979). Time of day and level of processing. Mem. Cognit..

[97] Folkard S, Monk TH (1980). Circadian rhythms in human memory. Br. J. Psychol..

[98] Dunne MP, Roche F, Hartley LR (1990). Effects of Time of Day on Immediate Recall and Sustained Retrieval from Semantic Memory. J. Gen. Psychol..

[99] Testu F, Clarisse R (1999). Time-of-day and day-of-week effects on mnemonic performance. Chronobiol. Int..

[100] Gorfine T, Zisapel N (2007). Melatonin and the human hippocampus, a time dependant interplay. J. Pineal Res..

[101] Martin B, Buffington ALH, Welsh-Bohmer KA (2008). & Brandt. J. Time of day affects episodic memory in older adults. Aging, Neuropsychol. Cogn..

[102] Wright KP, Lowry CA, Lebourgeois MK (2012). Circadian and wakefulness-sleep modulation of cognition in humans. Front. Mol. Neurosci..

[103] Maylor EA, Badham SP (2018). Effects of time of day on age-related associative deficits. Psychol. Aging.

[104] Puttaert D, Adam S, Peigneux P (2019). Subjectively-defined optimal/non-optimal time of day modulates controlled but not automatic retrieval processes in verbal memory. J. Sleep Res..

[105] Lang, P. J., Bradley, M. M. & Cuthbert, B. N. International Affective Picture System (IAPS): Instruction manual and affective ratings, Technical Report A-8. Gainesv. Cent. Res. Psychophysiology, Univ. Florida. (2008).

[106] Macmillan, N. A. & Creelman, C. D. Detection Theory A User’s Guide. Cambridge Univ Press New York. (1991).

[107] Fair DA (2008). The maturing architecture of the brain’s default network. Proc. Natl. Acad. Sci..

[108] Tzourio-Mazoyer N (2002). Automated anatomical labeling of activations in SPM using a macroscopic anatomical parcellation of the MNI MRI single-subject brain. Neuroimage.

[109] Diedenhofen B, Musch J (2015). cocor: A comprehensive solution for the statistical comparison of correlations. PLoS One.

